# ‘It’s where I belong’: what does it mean to age in place from the perspective of people aged 80 and above? A longitudinal qualitative study (wave one)

**DOI:** 10.1186/s12877-024-05139-2

**Published:** 2024-06-17

**Authors:** Kate Gibson, Katie Brittain, Emma McLellan, Andrew Kingston, Heather Wilkinson, Louise Robinson

**Affiliations:** 1 Population Health Sciences Institute, Health Innovation Neighbourhood, Newcastle University, Newcastle upon Tyne, UK; 2 Advanced Care Research Centre, Edinburgh University, Usher Building, Edinburgh Bioquarter, UK

**Keywords:** Ageing in place, Very old, Oldest old, 80+, Home, Economic resources, Social connections, Qualitative research

## Abstract

**Background:**

Most people want to remain at home as they age. Ageing in place – remaining at home and connected to the community – is a national and international policy priority; however, to better understand how policy might be implemented, a more nuanced understanding is required about older adults’ lived experiences of ageing in place, especially the experiences of those aged 80 and above.

**Objective:**

To describe and explore the social processes which enable ageing in place from the perspective of community-dwelling older people (80+).

**Methods:**

Forty-six respondents (80–100+ years) participated in the first wave of a longitudinal qualitative study set in North East England. Semi-structured interviews were conducted in participants’ homes between June 2022 and January 2023. Interviews were analysed using reflexive thematic analysis.

**Results:**

Participants positioned their homes as a place of freedom and as the antithesis of a ‘care home’. Remaining in place was important for all participants; a key priority for them was to remain physically active to enable this. However, many participants faced significant hurdles to remaining in place. These were primarily related to health and mobility issues. Some participants were able to overcome such barriers by drawing on financial resources and available social networks.

**Conclusion:**

The home is central to understanding older peoples’ (80+) experiences of ageing. In a socio-political context which promotes ageing in place, the social factors shaping experiences of ageing in place must be considered. This involves attending to the challenges of later life, particularly health and especially mobility and physical function. Currently, those with resources (social and economic) are better equipped to respond to such challenges, thus potentially exacerbating widening inequalities in ageing. By foregrounding the perspectives of those ageing in place alongside social factors shaping their experiences, our study has important implications for policy and health and social care. We show that a more equitable allocation of resources is vital to fulfil the ageing in place policy agenda. Furthermore, we highlight a need to recognise commitments to ageing in place displayed by people aged 80 and above, especially when remaining in place becomes difficult to achieve.

## Introduction

The agenda to create age-friendly environments has gained traction in recent years, most notably driven forward by the World Health Organisation [1]. A key component of the World Health Organisation’s framework for age-friendly cities and communities is an emphasis on ageing in (the right) place, – ‘to live in one’s own home and community safely, independently and comfortably, regardless of age, income or capacity’ [2](p.vii). A commitment to supporting older people to age in place underpins UK national and international health and social care policy [3, 4]. There are also significant economic arguments underpinning such policies, namely that ageing in place is understood to be a cost-effective means of managing the health and social care needs of the ageing population, of which in the United Kingdom 2.5% is over 85 years old, projected to rise to 4.3% by 2045 [5]. To ensure the maximum effectiveness of ageing-in-place policies, we need a deeper, more nuanced understanding of the social factors shaping ageing in place from the perspectives of older people themselves. Older people, especially those in later older age (80+),^1^ have been historically excluded from health research [7, 8] despite being more likely to use health and social care [9] and the fastest growing cohort of the population [10].

The guiding principle of ageing in place is that remaining at home, or in a familiar setting and connected to the community, fosters in older people an ‘increase in well-being, independence, social participation and healthy ageing’ [11] (p.220). Ageing in place is a preference emphasised by many older people themselves, many of whom spend a considerable amount of time at home and in their local neighbourhoods [12], especially those in later older age [13]. Research has established that the home can provide a sense of familiarity, independence, safety, freedom and identity while also linking people to neighbourhoods and communities [11, 14], in turn connecting older people to caring relationships that occur in those places [12]. By contrast, transitions from home to residential care are often negatively associated with loss of autonomy, freedom and privacy [15], increasing risk of loneliness and isolation [16], thereby compromising identity and belonging [17]. ‘Home’, therefore, is pivotal in ageing narratives [18].

The Newcastle 85+ study – a longitudinal cohort study exploring the health and wellbeing of older people aged 85 and over for ten-year period – establishes that increasing numbers of people reside at home in later life, often also living with multiple long-term conditions [19], but that at the same time declining mobility poses challenges to remaining in place [20]. There is a need for qualitative research to explore *how* this cohort negotiate declining mobility to remain at home. Ageing in place is not uniformly experienced; experiences are heterogeneous and fluctuate according to personal circumstances and shifting socio-cultural contexts [21, 22]. Furthermore, while a recent systematic review about middle aged and older people’s perspectives of their living environment established the importance of independence and autonomy associated with ageing in place [23], besides we know relatively little about the lived experiences or the processes enabling ageing in place from the perspective of older people themselves [13, 21], especially those in later older age [19, 24]. Given that ageing in place is a policy priority, qualitative research which incorporates the voices of people in later older age, the fastest growing cohort of the population [10], is vital to establish whether the idealisation of ageing at home reflects the lived reality. This paper focuses on cohort of older adults aged between 80 and 100+ residing at home without major functional impairments or without age-related formal care, a hitherto under-researched area [24], to provide an understanding of the nuances of social context and the role of resources in facilitating ageing in (the right) place. To this end, our aims are twofold. Firstly, we describe how a cohort of older adults aged 80 and above conceptualise home; secondly, we explore some of the social processes which shape their experience of living at home.

## Methods

This paper reports findings generated via the first wave of fieldwork (semi-structured interviews) from a longitudinal qualitative study aiming to examine how ageing in place is experienced and how such experiences change over time from the perspectives of a cohort of individuals aged 80 and above. The ongoing longitudinal study, based in North East England, is comprised of three waves of in-person interviews and interim participant observations conducted during home visits over three years. It is part of a wider cohort of studies hosted within the Advanced Care Research Centre.

Forty-six participants were recruited via primary care. Participants were purposively sampled to ensure representation across categories of gender and age. Community-dwelling individuals, with capacity to consent, and aged 80 years or older were invited to participate. Based on these specifications, we asked healthcare teams at three GP practices to screen patient lists to identify a cohort of eligible patients evenly distributed across gender and age and to include representation of ethnically minoritized individuals. Initially, each practice posted information packs to 18–24 patients (66 patients in total). From this first wave of recruitment, we secured participation from 20 participants. We then requested the healthcare teams each distribute information packs to a further 20–30 participants (74 patients in total), recruiting a further 26 participants. Information packs invited patients to telephone the research team or return a contact consent form in a self-addressed envelope to register their interest. Healthcare teams conducted follow-up telephone calls with patients who had not responded to ascertain their interest in the study and, if interested, elicit verbal consent to share the patient’s name and contact details with the research team.

Ethical approval for this study was obtained from the NHS Research Ethics Committee (reference 22/YH/0073). Participants were sent information sheets and provided written informed consent prior to interviews.

Interviews were conducted by KG and EM between June 2022 and January 2023 within participants’ homes (except one, conducted via video call). Interviews lasted an average of 61 min (range: 38–108 min). Interviews were comprised of open-ended questions and explored participant demographics, living arrangements, housing needs, care aspirations, and factors impacting on their experience of home. Interviews were audio-recorded and transcribed verbatim. Participant identifiers were anonymised following transcription and transcripts checked by the research team for accuracy. All names used here are pseudonyms.

Informed by an interpretative methodological approach, a reflexive approach to analysis was undertaken throughout data collection. Post-interview summaries written by EM and KG following each research visit documented observations of interview settings (for instance, participants’ homes and local areas) as well as aspects of the research encounters which could impact on later interpretation and analysis (for instance, interviewer-participant rapport). KG used these post-interview summaries to update a reflexive journal which as well as identifying empirical patterns, situated interpretations alongside existing ageing-in-place literature, acting as a starting point from which to conduct in-depth reflexive thematic analysis. Reflecting the values of the qualitative paradigm, reflexive thematic analysis not only acknowledges but also centres the researcher’s role in the production of knowledge. A process of deep reflection underpins the iterative development of analysis entailing ‘a continual bending back on oneself – questioning and querying the assumptions we are making in interpreting and coding the data’ [25] (p.594).

On completion of fieldwork, to allow for full immersion in the data, transcripts were re-read and, with the assistance of NVivo 12, line-by-line coding was conducted to allocate the entire dataset into descriptive codes (for example, the home, aids and adaptations, social relationships). Guided by post interview summaries, the reflexive journal and further reading, these codes were developed, reviewed, and then refined into substantive themes [26]. A focus on capturing participants’ perspectives and experiences of ageing in place was maintained throughout. For example, during the coding of the data a recurrent pattern of distancing from care homes was apparent within participants’ evaluations of their homes, which we conceptualise as our first theme (Home is everything, except for a care home) below. This iterative process of reflection, coding, cross checking, and analysis was discussed within the wider qualitative research team (KG, KB and EM) throughout, ensuring the rigour and validity of data interpretation.

**Table 1:** Participant Characteristics

		MALE (*n* = 20)		FEMALE (*n* = 26)		ALL (%)		
**Age**								
	80–84	7		11		18 (39.1)		
	85–89	10		8		18 (39.1)		
	90–94	3		4		7 (15.2)		
	95–99	0		2		2 (4.3)		
	100+	0		1		1 (2.2)		
**Ethnicity**								
	White British	20		24		44 (95.7)		
	South Asian	0		1		1 (2.2)		
	White Other	0		1		1 (2.2)		
**Time in neighbourhood**								
	0–9 years	0		1		1 (2.2)		
	10–19 years	0		1		1 (2.2)		
	20–29 years	4		4		8 (17.4)		
	30–39 years	2		3		5 (10.9)		
	40–49 years	4		2		6 (13.0)		
	50–59 years	4		9		13 (28.3)		
	60–69 years	2		3		5 (10.9)		
	70–79 years	1		0		1 (2.2)		
	80 + years	2		2		3 (8.7)		
	Unanswered	1		1		2 (4.3)		
**Household composition**								
	Lives alone	1		0		1 (2.2)		
	Lives alone (widowed)	6		15		21 (45.7)		
	Lives with spouse	12		11		23 (50.0)		
	Lives with spouse and adult-aged son	1		0		1 (2.2)		
**Housing Status**								
	Owned	17		20		38 (82.2)		
	Rental (Private, council or social housing)	2		5		7 (15.5)		
	Owned by non-resident family member	0		1		1 (2.2)		
**Indices of Multiple Deprivation (IMD) Quintile** ^**a**^								
	1 (most deprived)	3		3		6 (13)		
	2	4		7		11 (23.9)		
	3	3		3		6 (13)		
	4	8		6		14 (30.5)		
	5 (least deprived)	2		7		9 (19.6)		
**TOTAL**		**20**		**26**		**46**		

^**a**^ The IMD combines information from seven domains (including income, health, employment, and crime) to produce an overall measure of deprivation for small areas in England. Quintiles are calculated by ranking small areas across England from most deprived to least deprived. Areas in Quintile 1 are in the 20% most deprived and areas in Quintile 5 are in the 20% least deprived

### Participant characteristics

Participant characteristics are presented in Table 1. At recruitment, participant ages ranged from 80 to 100+. Although the sample was evenly distributed across gender, age, and household composition, most participants were white homeowners and almost half of the sample resided in areas characterised as less deprived according to the IMD. The lack of ethnic diversity reflects the predominantly white population in North East England in this age group [27]; the disproportionate weighting towards homeowners reflects the high proportion (79%) of older homeowners in the UK [28]. Some participants reported having no health issues; others reported having multiple long-term conditions including arthritis, macular degeneration, Type 2 diabetes; two participants had recent dementia diagnoses. One participant was visited by formal carers at home.

### Findings

Findings are grouped into the following interrelated themes:

1. Home is everything, except for a care home.
2. Remaining active to remain at home.
3. Hurdles to remaining in place: health and mobility issues.
4. Overcoming barriers to ageing in place: financial resources and social networks.

Below we present a summary of these themes, supported by participant quotes. Quotes are contextualised with demographic information, presented as follows: pseudonym_gender_age_IMD Quintile (IMD).

### Home is ‘everything’, except for a care home

Regardless of social location, the home was important across the dataset. It was associated with autonomy and independence; comfort and security; a link to the local community; and sometimes explicitly positioned as a ‘haven’:

*It’s my sanctuary, it’s my haven, it’s my safe place.* (Mary_F_85yrs_IMD5)

*It means everything to me. It’s my security, my happiness, everything is here.* (Beryl_F_85yrs_IMD5)

*It’s home. It’s where I belong. I love to take a break elsewhere. But this is where I fit. It’s tailormade.* (Alfred_M_86yrs_IMD4)

To use Alfred’s words, the home is ‘tailormade’ engendering a sense of belonging which is deeply congruent with his identity. The significant nature of this attachment to home was for many participants captured by a simple phrase, ‘*it’s everything*’:

*Well, it’s everything, isn’t it?* (George_M_90yrs_IMD1)

*It’s everything, really. I just want to die here.* (Ned_M_80yrs_IMD4)

Because of their home’s significance, participants wanted to remain there for as long as possible. While this was about attachment to place, for many, underpinning this sentiment was an explicit distancing from residential care. When discussing the value of home, many participants, without prompt, evoked a dichotomy between *their* home and *a* care home:

*When you’re at home, you eat what you like to eat. When you’re in a home, you eat what they give you. (Laughter) … When you’re at home, you get up when you want to get up, go to bed when you want to go to bed… …It’s the freedom of being able to do- And the lack of being regimented.* (Wilfred_M_89yrs_IMD4)

*I wouldn’t like to go into a care home for instance, I just want to stay here as long as I can. I cut my own grass, do my own weeding and…* (John_M_89yrs_IMD2).

*These care homes, you stagnate there. People stick you in there because they can’t be bothered to look after you* (Mabel_F_81yrs_IMD5).

Home, associated with autonomy, comfort, and independence, was the antithesis of residential care.

### Remaining active to remain at home

A commitment to continued independence in place was valued and prioritised across the sample. For many participants a positive attitude was a factor in their success:

*I will go, go, yes and I never give in, no, I don’t believe in that* (Fred_M_82yrs_IMD4).

An attitude of ‘not giving in’ underpinned participants’ commitments to remaining active in order to avoid declining mobility. Notably, several participants saw the home itself as providing a context to pursue physical activity:

*As long as my legs can carry my weight, it’s better if I move around in the house. Otherwise, if I sit, I will be sitting forever.* (Ida_F_90yrs_IMD2)

*You mustn’t give in. It is fatal. If you can possibly get away with making yourself do it. And the heart people like me to pedal. I have a static bike out there which is totally boring, but I do try and knock off a hundred every now and again, because the idea is if you let your leg muscle go you are in trouble.* (Isobel_F_100+_IMD3)

Significantly, from subtle movements to regular exercise, participants undertook a range of activities to prolong mobility, even when faced with sometimes significant health-related adversities. Many participants avoided mobility aids and adaptations to remain active, despite having a clear need:

*Somebody said once about getting an electric scooter, and I said, “No, I don’t want to.” I think once you give in to not walking at all* (Edna_F_88yrs_IMD4).

*The stairs are good exercise. People say, “Why don’t you have a stairlift?” I say, “What for? If I don’t use my legs, I will lose them.”* (Sally_F_81yrs_IMD4).

This aversion to using aids and adaptations, justified with a ‘use it or lose it’ attitude, is evidence of participants’ awareness of the need to remain active and mobile to remain in place, but also the ways in which their home, and its perceived associations with independence, becomes a setting to pursue such activities. However, despite this sample-wide commitment to remaining active, for some, health and mobility issues impacted on the extent to which home was in fact a haven for safety and independence.

### Hurdles to remaining in place: health and mobility issues

For some participants, despite their positive attitude and their commitment to remaining active at home, health and mobility issues significantly impacted on their everyday experience of home:

*The stairs, I find very, very, arduous now… …As far as the kitchen is concerned, the problem I have now is I can’t reach things. We’ve got quite tall cupboards but, because I’ve got limited movement in this arm as well* (Emily_F_84yrs_IMD4).

*I’m not well enough to do anything in the garden. Even stooping tires me a lot* (Wilfred_M_89yrs_IMD4).

*I used to do my own washing and hang it out, but I can’t do that now, so I just hang it in the bedroom, you know… …dusting the photographs, it’s hard stretching up* (George_M_90yrs_IMD1).

*If I get the bin out- sometimes I take [Name]’s up, our nephew’s up as well. I’ll take one out, but I’ll only get halfway with the next one because of my spine. I just tighten up with the arthritis, it just bites in, so that’s only about 50 yards.* (Ned_M_80yrs_IMD4)

Mobility issues, then, were for some participants fundamental barriers to achieving ongoing independence at home. As the excerpts suggest, this is related to the physical layout of the home (for example, the stairs, kitchen, and garden), undertaking home maintenance activities (for example, dusting and washing) and enacting neighbourly activities (such as taking the bins out). Furthermore, declining mobility could impact on the quality and frequency of social connections beyond the home, at worst result in an inability to leave the house at all:

*I’d like to go back to town to shop because it’s so useful. You know, you can visit the bank and do all that’s necessary. But I don’t feel as confident, now, on my legs.* (Emily_F_84yrs_IMD4)

*If I could get out, I would go out*’ (George_M_90yrs_IMD1).

### Overcoming barriers to ageing in place: financial resources and social networks

Having access to financial resources and/or social networks helped alleviate some of the limitations imposed by declining mobility and ill-health.

#### Social networks

Despite many participants living with health and mobility issues, at the time of interview only one participant, Ida, was visited by formal carers. Instead, participants highlighted the caring roles of family, friends, and local people.

Family members, most often children or grandchildren, played an integral part in many participants’ experiences of home. Care-related activities included providing transport, checking in, and undertaking domestic chores:

*My children come and they prepare something for the evening* (Ida_F_90yrs_IMD2).

*She [daughter] comes two or three times a week, but she phones every morning at 9 o’clock, and every evening at about 7:00pm to make sure I’m alright* (Tommy_ M_84yrs_IMD1).

For those living with spouses, being in a pair facilitated ageing in place, especially – as indicated by the quote below – by managing risk:

*When we shower there’s always two of us, one in the bedroom and one showering. We stay. When he showers, I’m there; when I shower, he’s there. We do things like that, we don’t risk.* (Irene_F_90yrs_IMD5)

Many participants also described feeling ‘looked out for’ in the community:

*People hail me all the way up the road, and we stop and have a chat – “How are you doing today?” It makes all the difference to life.* (Alice_F_92yrs_IMD3)

*Next door, we’ve got a system. There’s a little window at the back, if that blind’s not up, if those blinds stay down by 11:00, she’s got a key.* (Derek_M_83yrs_IMD3)

#### Financial resources

Some participants could deploy financial resources to sustain their homes, or in some instances create a new home, to accommodate their ageing needs.

Participants employed gardeners and/or cleaners to undertake difficult domestic tasks, in turn supporting them to maintain their homes and gardens:

*I pay a gardener to come, to do the gardening. I’m very fortunate that I’m able to do this, not everybody can afford to pay.* (Alice_F_92yrs_IMD3)

*What they [the cleaners] do is a good hoover and they can move the bits of furniture and things like that. And if I’m stuck, like when I had my shoulder, they changed the beds, and they took that away and did the laundry and brought it back* (Mary_F_85yrs_IMD5).

Some participants had paid for adaptations including wet rooms, reclining beds, bath lifts, grab rails, and stairlifts, while others had ‘downsized’, relocated and even purpose-built age-friendly homes. Factors guiding these decisions centred on ensuring the physical layout as well as geographic location could accommodate participants’ ageing needs:

*When we built the bungalow, one of our bedrooms has a wet room and a sink that you can put a wheelchair under it, with the thought of not wanting to move out of here other than feet first* (Grace_F_81yrs_IMD2).

*Always in my head was, “As I get older, I want to be in a one storey house where there are no stairs.” So, that’s what I was looking for, for myself. Then, obviously, as you get older, you’re beginning to think about the amenities. What is available, close to you? Where’s the bus stop? Where’s the hospital? Where’s the doctor?* (Evelyn_F_97yrs_IMD5)

Evidently, deploying financial resources to adapt, maintain, and create environments allows for the continuation of what is valued about ‘home’.

## Discussion

This paper’s principal objective was to explore ageing in place from the perspectives of a newly recruited cohort of 46 community-dwelling adults aged 80 years old and over. In so doing, it responds to calls for further insight about processes which shape older people’s experiences of ageing in place [13, 21], especially those of adults in later older age [19]. Across the sample, participants were committed to remaining at home, a place associated with security, independence and belonging. A hostility towards residential care, in particular its alignment with (institutionalising) loss of autonomy and independence, emerged as highly significant. Participants sought to mitigate the possibility of transition to residential care by prioritising remaining active, often despite physical limitations. Importantly, having access to financial resources and/or social connections helped alleviate some of the limitations imposed by declining mobility on remaining in place.

Our finding that home is valued for providing a sense of independence, security, and belonging is consistent with research exploring the home-based experiences of people in later older age [11, 13, 29], much of which focuses on residential transitions [15, 17, 19, 30]. Our findings demonstrate that an abject image of residential care occupies a significant place in evaluations of home for people aged 80 and above. Older people’s hostility towards care homes has been alluded to in previous research [13, 31]. Significantly, our participants often directly juxtaposed their understandings of home with residential care, and an aversion to care homes emerged as an equal, if not more, important motivator for staying at home. Given the age range of our participants, and related risk of frailty, a heightened awareness of impending loss of independence is perhaps not surprising; a proximate need for formal care (residential or not) is a likely possibility. As noted elsewhere, ‘home becomes a last bastion, a bulwark of resistance against unwelcome change, and the losses that accompany it’ [13](p.15).

Participants’ commitment to remaining active resonates with research identifying the importance of purposeful, adaptive, and incidental activities to older people [32], in particular insights that spatial and physical contexts of homes are sites to pursue healthy ageing for people in later older age [29]. The community-dwelling older adults in Larssen et al’s study prioritised being occupied through ‘everyday doings’ to maintain cognitive and physical health and maintain independence [24]. Similarly, in our study we show how commitments to physical activity centred on both retaining independence in place and delaying loss of function, as evidenced by the ‘use it or lose it’ attitudes underpinning the avoidance of relying on mobility aids for example. Remaining active emerged as a means of retaining personal control and autonomy, identified previously as integral to the psychological wellbeing of those in later older age as they increasingly face possible transitions, adaptations, and personal losses [13]. While our findings elucidate the various activities undertaken by our participants to remain at home, as everyday activities inevitably become more challenging, home becomes less of a ‘comfortable safe haven’ and more of an obstacle to overcome. To this end, increasingly difficult domestic practices, especially those concerned with maintaining home (such as cleaning and washing), threaten to both rupture idealised notions of home, and bring older peoples’ capacity to stay there into question by signalling a need for formal care [33]. If some of the challenges older people experience undermine independent living, the reality of ageing in place is, for some, in contrast with the ideal [11].

Hatcher et al. call for more research into the strategies and ‘personal qualities’ that older people draw on to remain at home [14]. While we show how ‘not giving in’ underpins our participants’ strategies for remaining active in place, our insights move beyond such individualised understandings. By locating experiences in a broader social context, we show how some participants could deploy financial resources and harness available social connections, such that barriers of declining mobility and ill-health were not always insurmountable. Community connectivity and social networks are important factors for ageing in place [14, 21, 31, 34, 35], and these attachments fluctuate over the life-course [21]. This is inadequately explored in relation to those in later older age [13]. We identify the integral role of families and friends in supporting those in later older age to remain at home. We also note that several participants encountered ‘incidental’ care in, sometimes unlikely, community settings [36]; however, declining mobility and ill-health poses a risk to such connections.

Extending literature which establishes that financial instability negatively impacts ageing in place [29, 37, 38], here we demonstrate how access to financial resources *enables* ageing in (the right) place. Several participants could renegotiate their home by deploying financial resources. Home maintenance is crucial for ageing well in place [33, 39], and as noted elsewhere [15, 33], many participants sourced paid-for services to undertake home maintenance tasks, a level of autonomy those without financial resources can ill-afford. Other participants adapted, relocated, downsized or purpose-built homes to better accommodate their ageing needs. We know that older people want to make choices about where they age [35] but that inequalities exist between those able to decide where they live and those marginalised and alienated by their communities [40]. Our findings demonstrate the role of financial resources in enabling autonomy and choice to create an age-friendly environment. By contrast, the few participants residing in social housing – well known for poor living conditions and correlated with the early onset of care needs [41, 42] – did not speak of such advantages. They were not able to exercise such autonomy and choice regarding their living environment. Instead, they were reliant on unpaid carers, well-known to be at heightened risk of declining physical and mental health as a consequence of being carers [43]. Having limited financial resources is a barrier to remaining in place [38], and given that caregiver burden is a significant risk factor for care home transition [44], it is our contention that our participants in precarious financial positions and solely reliant on informal care are at increased risk of transitioning into residential care. Although further longitudinal research is required to explore the nature of such possible transitions and how they may be experienced. Many of our participants are at the cusp of needing formal care, although at present are sustaining their needs (and homes) via informal arrangements, such as family members or by employing gardeners or cleaners. Future research would usefully explore how formal care is deferred and if not, how it emmeshes into the informal care networks participants have in place.

.

### Limitations

This study is limited by its narrow geographic focus, and the homogeneity of our sample (comprised primarily of white homeowners). Likewise, the perspectives of isolated older people are missing from this research. While the lack of diversity in our sample has implications for the transferability of our findings, its homogeneity has allowed for deep insight about the role of resources in *facilitating* ageing in (the right) place. Nevertheless, future research would benefit from the inclusion of ethnically and economically marginalised groups to scrutinise how experiences of ageing in place are intersected by social categories such as class and ethnicity. Furthermore, there has not been scope in this paper to explore nuances relating to gender and age range across the cohort as shaping participants’ attachments to place and their ability to remain there. Notwithstanding these limitations, a key strength of our study is by giving primacy to the voices of people aged 80 and above, we have foregrounded their perspectives and experiences in context. In a rapidly ageing population, it is vital that the voices of older people inform the development of policy and practice; without their insight, efforts to support the population to age in place are likely to be suboptimal.

## Conclusions

Healthy ageing is a global priority [45]. It is, in policy, emphasised as attainable through increased activity, independence, autonomy, and community integration, a self-responsibility which our participants internalised regardless of the social and physical environments which shape such outcomes. Currently, ageing-in-place policy allocates the responsibility for care to local communities, individuals, and informal care networks [46]. Without significant upstream intervention, those without access to resources are unfairly disadvantaged. Fulfilling the ageing-in-place agenda requires that homes and communities are age-friendly. But a combination of local austerity measures and the aftermath of COVID-19 has meant reduced investment into local community infrastructures [47]. Local authority allocations of home adaptations, for instance, are vastly insufficient for the needs of the ageing population [48]. This, together with a steady decline in state-funded social care, has meant that informal carers are disproportionately burdened with the emotional, physical and economic costs of caring [43]. Those with access to resources can better mitigate this impact. Without more equitable allocations of resources to individuals and communities, investing into homes and local neighbourhoods to ensure they are accessible and age-friendly, and strengthening local support networks, ‘ageing in (the right) place’ [2] will remain accessible to a limited few. At a time of economic austerity, this presents a significant challenge to policy makers.

In addition to policy, our qualitative enquiry has important implications for health and social care practice. Physical activity interventions targeting older people predominately follow ‘behaviour change’ modelling [32], most notably to reduce ‘sedentary behaviour’ [49]; however, less is known about *how* initiatives can encourage physical activity [10]. We suggest interventions focus less on behaviour change and more on attending to the underlying social, physical, and economic factors shaping orientations to physical activity. Contextualising activity in this way means acknowledging the deep commitments to prolonging independence displayed by our participants through their attitude to remaining active. Participants were well-aware of the benefits of physical activity and prioritised remaining active at home, itself a social and physical environment valued for providing a sense of safety, independence, and autonomy. Thus, we also echo recommendations that initiatives should incorporate physical and social context to support older people to undertake everyday *unstructured* physical activity in their homes [32]. These should be personalised and tailored to individual physical limitations and living environments rather than a one-size-fits-all approach. While in times of economic uncertainty this poses a challenge, if such initiatives facilitate ageing in place, in the longer term they will likely decrease financial expenditure by reducing formal care needs or delaying transitions into residential care. Finally, regardless of their commitments to ageing in place, several participants will likely require formal care or transition into residential care. Given their heightened sensitivities towards residential care, it is imperative that health and social care professionals involved with supporting older people to stay at home or indeed organising transitions to care homes, recognise the sentiments underpinning attachments to place, even if place appears unsafe. This is not to suggest that we advocate for older people to remain in unsafe environments, but rather that the stigma of requiring formal care is directly addressed if because of such stigma older people remain in unsafe homes. When policy aligns independently remaining in place with ageing well, then due consideration must be paid to the adverse psycho-social consequences to older people when this becomes difficult to achieve.

## Data Availability

The datasets generated and analysed during the current study are not publicly available due to reasons concerning participant privacy and confidentiality. Please contact the corresponding author (kate.gibson2@newcastle.ac.uk) for further information.

## Footnotes

1 The terms ‘very old’ and ‘oldest old’ are commonly used to refer to those over the age of 80; however, following insight that such terminology can have ageist connotations [6], we have avoided using these terms.
